# The implementation realities of a digital antenatal care improvement intervention: Insights from ethnographic work in primary health facilities in Nepal

**DOI:** 10.1371/journal.pdig.0001340

**Published:** 2026-04-06

**Authors:** Sulata Karki, Seema Das, Emma Radovich, Abha Shrestha, Rajani Shakya, Abha Shrestha, Ona L. McCarthy, Oona M. R. Campbell, Biraj Man Karmacharya, Loveday Penn-Kekana

**Affiliations:** 1 Dhulikhel Hospital Kathmandu University Hospital, Research and Development Division, Dhulikhel, Nepal; 2 London School of Hygiene and Tropical Medicine, Faculty of Epidemiology and Population Health, London, United Kingdom; 3 Kathmandu University School of Medical Sciences, Department of Community Medicine, Dhulikhel, Nepal; 4 Kathmandu University School of Medical Sciences, Department of Obstetrics and Gynecology, Dhulikhel, Nepal; 5 Kathmandu University School of Medical Sciences, Department of Public Health and Community Programs, Dhulikhel, Nepal; Yonsei University College of Medicine, KOREA, REPUBLIC OF

## Abstract

Nepal has significantly improved maternal health service coverage, including antenatal care (ANC), with a growing focus on quality care. The mobile health Integrated Rural Antenatal care (mIRA) project evaluated an electronic decision support system (EDSS) to improve ANC quality in primary healthcare facilities. This sub-study was undertaken within the context of a mixed-method evaluation. It aimed to explore how auxiliary nurse midwives (ANMs) implemented the EDSS and to provide insight into the real-world challenges and processes of the mIRA implementation project. We conducted a focused ethnography in four primary health facilities, where ANMs implemented the EDSS. Data were collected between December 2021 and January 2023 and involved repeat ethnographic observations, informal conversations, formal interviews (n = 16), and validation workshops for participants to respond to and reflect on findings. The data were analyzed thematically, drawing on street-level bureaucracy theory. ANMs verbalized they valued the EDSS, but this was not reflected in their use of it. ANMs adopted the EDSS primarily for recordkeeping and did not comply with care recommendations that conflicted with their usual way of working. ANMs viewed themselves as providing care for normal pregnancies and believed that quality was not an issue, as they felt they were already delivering good care at their level. This perception, coupled with a risk-averse attitude, led them to refer cases to doctors or higher centers rather than implement the EDSS recommendations. The EDSS recommendations to improve the quality of ANC did not align well with ANMs’ usual practices, which were influenced by contextual factors and the complexities of organizing ANC in primary health facilities. By triangulating longitudinal data collected through multiple methods, the study provided a comprehensive insight into ANC provision in Nepal, revealing the challenges associated with improving quality, unpacking the realities of intervention implementation, and highlighting the disconnects between rhetoric and practice.

## Author summary

The mobile health Integrated Rural Antenatal Care (mIRA) project tested an electronic decision support system (EDSS) in primary healthcare facilities to guide and support auxiliary nurse midwives (ANMs) in providing better antenatal care. As part of this, we conducted an ethnographic study in four facilities to examine how the EDSS was used in daily practice. Data were collected through observations, informal conversations, interviews, and workshops where participants could reflect on their experiences. We found that ANMs valued the EDSS but mainly used it for recordkeeping rather than following care recommendations prompted by the application. They considered themselves already capable of providing good care for normal pregnancies and tended to refer mildly complicated cases to doctors or higher-level facilities instead of following the EDSS. The perception that they were already providing good care, their routine practices, their risk-averse attitudes, and the contextual realities in which the EDSS could not fit into their usual system of providing antenatal services all influenced how the EDSS was used. This study highlights the challenges of introducing digital tools and demonstrates how ethnography can reveal the realities of implementing quality improvement initiatives through EDSS.

## Introduction

Globally, maternal and newborn mortality has decreased in recent decades; nonetheless, disparities in pregnancy outcomes persist, with the majority of deaths occurring in low- and lower-middle-income countries (LMICs) in Sub-Saharan Africa and South Asia [1]. In Nepal, maternal and perinatal mortality remains high, with an estimated 142 maternal deaths per 100,000 live births [2] and 13.5 stillbirths per 1000 total births in 2023 [3]. Nepal has achieved substantial improvements in maternal health service coverage, with 81% of women attending four or more antenatal care (ANC) visits and 79% of births taking place in facilities, according to the 2022 Nepal Demographic and Health Survey [4]. However, increased service utilization alone is not sufficient for achieving good health outcomes; improving quality of care, including for ANC, is critical to preventing maternal and perinatal deaths [5–7].

Nepal’s Roadmap for Maternal and Newborn Health emphasizes quality improvement, highlighting persistent barriers within health system, such as inadequate human resources, limited infrastructure, and accessibility challenges that hinder the delivery of high-quality, accessible maternal health services [8,9]. As part of this strategy, the Government of Nepal has sought to strengthen ANC quality through adoption of the World Health Organization’s (WHO) recommendation of eight protocol visits during pregnancy [8,9]. At the time of this study, the nationwide rollout of eight protocol ANC visits was underway.

Digital health interventions, particularly electronic decision support systems (EDSS), have been promoted as tools to improve quality of care via better adherence to clinical guidelines and supporting healthcare providers’ decision-making in resource-limited settings [10–12]. Evidence suggests that EDSS can improve specific care processes; however, findings from LMIC contexts are mixed, and implementation challenges are frequently reported [10,13]. Factors related to the process of EDSS implementation, including the organizational context, are often poorly investigated, despite being important to implementation and intervention success [14–17]. Studies focused on clinical outcomes or guideline adherence, often offer limited insight into how EDSS are used in everyday practice, how frontline providers engage with them, or how health system and sociocultural contexts shape their implementation, uptake and effectiveness [10,13,18]. Contributing to this evidence gap can help advance the design and implementation of EDSS interventions aiming to improve the quality of maternal health services in LMICs.

In Nepal, the mIRA (**m**obile health **I**ntegrated **R**ural **A**ntenatal care) project implemented two Android tablet-based EDSS, the mIRA EDSS [19,20] and the WHO EDSS [21] that sought to improve ANC quality. Briefly, both EDSS were designed to be used by ANC staff or auxiliary nurse midwives (ANMs) throughout all stages of pregnancy, offering tailored care prompts for different gestational ages, identifying missing care components, responding to screening outcomes or vital signs input, and providing counselling advice [19]. The mIRA EDSS was adapted to align with Nepalese ANC guidelines [22]; the interface included navigation menus and data entry fields for history, examination, laboratory results, and provided visual pop-up alerts for conditions such as anemia, pregnancy-related hypertension, and gestational diabetes, along with guidance on diagnosis and treatment [19]. The WHO EDSS supported ANMs in applying WHO ANC guidelines through checklists for routine care, screening, and referrals, but it did not offer guidance on managing pregnancy complications [23]. Both EDSS functioned offline and synchronized data when connectivity was available. The EDSS intervention, workflow and interface are provided in the supplementary file (see S1 Text). Detailed descriptions of the intervention, design and methods of the evaluation are available elsewhere [19,20,23–26].

The published evaluation of the mIRA project identified several implementation challenges, including inconsistent use of the EDSS, facility readiness constraints, inflexibility in aligning with ANC staff’s decision-making, mixed perceptions of EDSS benefits in daily use, and a lack of full integration into existing health systems [23]. However, these findings raise further questions about the day-to-day realities of EDSS implementation. Specifically, how frontline providers, particularly ANMs, incorporated EDSS into their routine work, navigated its constraints, and adapted it within existing clinical and organizational practices remains insufficiently understood. Our ethnographic study addressed this gap by providing insights into implementation realities, and in doing so, offers evidence to support the design and adaptation of digital interventions within ANC services. Ethnography is increasingly advocated and applied in evaluative studies due to its holistic approach and its strength in explaining how and why an intervention works or does not [27–29]. It has proven useful for examining the dynamics of complex interventions and their interactions with the systems of relationships and structures in which they are implemented [30].

By applying an ethnographic approach, we focused on the “context” and “daily lives” of ANMs working in the primary healthcare facilities. In this paper, our primary aim is to explore how ANMs implemented the EDSS and to understand the real-world challenges and processes that influenced its use in primary healthcare settings. We present key ethnographic findings that offer insights into the broader mIRA implementation project in Nepal [23] and reflect on how an ethnographic approach can inform future digital health interventions in maternal health.

## Methods

### Study setting

This study was undertaken within the context of a mixed-method evaluation, the overall results of which have been published elsewhere [23]. The mIRA project took place in 19 rural primary health facilities in Nepal [23]. We conducted a focused ethnography [31] in four government-run primary healthcare facilities selected from these 19 sites: two primary healthcare centers (PHCC) – one implementing the mIRA EDSS and one implementing the WHO EDSS, and two health posts (HP) – one implementing the mIRA EDSS and one implementing the WHO EDSS. These facilities were purposively selected because municipal officials considered them well-functioning, and they had the highest ANC volumes. These criteria were applied to capture insights from facility contexts thought to be well placed to implement and benefit from EDSS and thus offer insights into both successes and challenges encountered. In total, 16 ANMs used the EDSS across the four health facilities. Additionally, we chose these facilities located within a 3-hour travel distance from the research base at Dhulikhel Hospital, for practical and safety reasons. Researchers stayed in nearby accommodations at these sites.

### Data collection

Data were collected using a focused ethnographic approach, which is an approach commonly used in health systems and services research where multiple qualitative data collection methods are used over a set time period to generate rich insights of context, overcoming many of the limitations of single, cross-sectional methods of investigation [31,32]. We used overt ethnographic observation [33], informal conversations and formal interviews with facility staff, informal conversations with ANC clients, and workshops with ANC staff, facility managers, and other key stakeholders to respond to and validate initial findings. Before the start of the study, two Nepali researchers (SK and SD) had frequent online meetings with the study’s medical anthropologist (LPK), who provided expert guidance and supervision, to develop the study guide. Both researchers conducting the observations and interviews were qualified public health professionals with experience in qualitative research, and one is a trained nurse. The observation topic guide, along with the interview topic guide, structured the data collection around key research questions over the multiple visits to the study facilities (see S2 Text). We adapted the topic guides based on continuous discussions within the research team about emergent themes from the fieldwork.

We collected data from December 2021 to January 2023, making three visits to each of the four facilities, including a final visit for validating preliminary findings. The first visit was made before the implementation of the EDSS to collect baseline data and information. We visited each facility for two subsequent periods after EDSS implementation. In total, we spent around two months in a PHCC-level facility and one month in a health post-level facility across multiple time points. In this period, we also conducted a separate time-motion study (observing and recording time taken by ANMs to conduct ANC tasks) in the two PHCCs [19,24]. We had informal conversations and interactions with the facility in-charge and other staff, such as medical officers, laboratory staff, paramedical staff, and office assistants in all facilities during our field visits. Rough field notes and mental notes were taken every day during visits to the case study facilities. Notes were written up, in detail, in English at the end of each day. The two researchers discussed and reflected on each day of fieldwork and planned for the following day’s data collection. A Google Drive folder was organized to upload all the written field notes individually by SK and SD which were reviewed by LPK and ER. All the data and information written in the field notes along with other relevant documents were de-identified and shared with the research team. During and after each period of fieldwork, the research team debriefed and discussed what was observed, reflecting on emerging findings, where we had saturation emerging within and between facilities, where we had a range of views, what we needed to explore further and triangulating between data sources and across facility sites. The formal interviews were audio-recorded, and the informal conversations were noted in the field notes.

We (SK and SD) conducted formal, in-depth interviews with all ANMs at these facilities; 16 ANMs were interviewed at the end of the third visit (July to September 2022). The interviews were conducted in Nepali and lasted approximately one hour. All the formal interviews were audio recorded, transcribed into Nepali, and then translated into English. The English transcripts were reviewed against the audio recordings by SK and SD.

At the end of the fieldwork, we presented our preliminary findings to the staff at each case study facility to obtain their perspective on what we had found, ask for their assistance in confirming the findings, and seek feedback. We also aimed to provide explanation or further clarification on our findings. These meetings and the discussions that emerged were considered part of the research process. These validation meetings took place between November 2022 and January 2023.

### Data analysis

Data analysis was started during data collection; frequent debriefing meetings were held among the team (SK, SD, ER and LPK) to discuss impressions and reflections from the field visits. Complete field notes for one facility and a transcript of one interview were independently coded by three persons (SK, SD and LPK). SK, SD and LPK met face-to-face to discuss initial codes and agree on major lines of inquiry. Field notes and transcripts were re-read thoroughly and repeatedly. All the field notes and interview transcripts were coded and analyzed independently by SK and SD, using the software Dedoose 9.0.85, and emerging themes were shared with and discussed iteratively with the research team. During the analysis, discussion and similarities were noted with Street-Level Bureaucracy (SLB) theory [34], and this was used to help advance the analysis and structure the results. We also adapted the concepts of coping behavior by Tummers and colleagues [35] to inform themes and interpretation. Data displays (tables of themes and examples), using SLB theory to structure the results [35,36], helped guide the discussion of the findings and informed the final interpretation.

### Ethics approval

This study received ethical approval from the Nepal Health Research Council (Reg. no.: 200/2019) and the London School of Hygiene and Tropical Medicine (Ref: 25094). We acquired permission from the local municipalities in which health facilities were situated. Written informed consent was received from all the facility in-charges and all ANMs during the first visit. Interviews and validation workshop discussions were audio-recorded with the consent of ANMs and facility in-charges. The transcripts and field notes were anonymized. Additionally, we have omitted the name of the district and health facility in which the study was conducted to maintain confidentiality.

## Results

The results are organized into two sections. The first part describes the facilities where the study took place, how ANC care was organized, and how staff talked about and used the EDSS. The second part describes beliefs about the quality of ANC, drawing on SLB theory to consider whether the ANMs behaved in ways that benefited the ANC clients when implementing ANC guidelines and EDSS recommendations.

### Part 1: Antenatal service provision and discretionary practices shaping EDSS use

#### Service provision varied across primary level facilities.

The characteristics of the four participating facilities are summarized in Table 1, using data from the first visit, conducted between December 19, 2021, and March 15, 2022. However, a key finding in the ethnographic work – and which emerged in monitoring at other facilities in the mIRA project evaluation – was frequent change in the facilities. During the study, Facility A, was upgraded to a municipal hospital offering caesarean sections and Facility C provided laboratory testing and ultrasound scanning services. In Facility B, the local election resulted in temporary staff cuts and the re-distribution of ANMs to other departments; there was no medical officer toward the end of the study period in Facility D. Changes impacted how ANC care was provided in the facility and also EDSS implementation, with turnover of ANMs trained to use the EDSS, and ANMs sometimes preoccupied with other health priorities outside of maternal and newborn health. Despite purposively selecting clinics with higher client loads, there were relatively few clients accessing ANC at the four facilities. This impacted ANC service provision and implementation of the EDSS. ANC staff sometimes had long periods where they had little to do in ANC, when they either sat around chatting or were deployed to different service areas. Low client load resulted in staff reporting that they rarely saw clients with complications such as hypertension or gestational diabetes and had little confidence to treat them. If ANC clients were seen with any minor abnormalities (e.g., nausea, abdominal pain, slightly raised blood pressure, etc.), ANMs immediately referred to either the doctor at the facility or to a higher-level facility, reflecting risk-averse coping practices.

**Table 1:** Facility profile.

Facility characteristics	Facility A	Facility B	Facility C	Facility D
Level	PHCC*	PHCC	HP	HP
Facility in-charge	Medical Officer (MO)	MO	Health Assistant	Health Assistant
Designated ANC day	No	No	Tuesday^#^	No
ANC client load (min-max)	2-15 per day	1-12 per day	2-3 per day**	1-4 per day
Number of ANC staff	5 ANMs	3 ANMs and 1 staff nurse	5 ANMs	3 ANMs
Number of medical doctors	MDGP† (1), MO (3)	MO (3)	MO (2)	MO (1)
Total staff	26	23	13	9
Laboratory tests	Yes	Yes	No	Yes
Ultrasound machine	Yes	Yes	No	Yes (once a month)
Monthly number of deliveries	25-30	2-4	15-30	2-3

*Upgraded to municipal hospital during study period

# *ANC examinations offered on other days if clients arrived*

**8–35 (min-max) in ANC day

*†Doctor of medicine in general practice*

#### Organization of antenatal care as routinized and unsystematic practice.

Antenatal care was primarily provided by ANMs who received 18 months training on ANC service in primary health facilities. A staff nurse, who had completed three years training, was also present at one facility. ANMs were either appointed on permanent contracts (for which they had completed an additional national exam through public service commission) or on temporary contracts. ANMs were also responsible for providing delivery and post-natal care. Additionally, they were observed doing family planning, medical abortion, immunization, COVID-19 vaccination, support in the outpatient department, emergency care, medicine distribution, outreach clinics, home visits, nutrition services, recordkeeping, and reporting monthly health management information system data.

We frequently observed two or three ANMs working together to perform the ANC consultation for a single client. One ANM-one client consultation happened sometimes when an ANM was alone in the ANC room, such as when other ANMs were engaged elsewhere. Sometimes one or more ANMs dealt with two clients simultaneously (Box 1). This was mostly the case with Facility A.

**Box 1. Case example of three ANMs working together to perform ANC consultations for two clients simultaneously**

There was a bench stationed inside the ANC room where four clients could be accommodated. All ANC clients arriving at the facility, entered the room directly and sat on the bench. There were three clients inside the room for the ANC check-up. ANM B, ANM C, and ANM D were working together. ANM B started asking about the medical history of client 1 after receiving woman’s handheld card. ANM D was examining client 2. She then called the client to lie down on the bed and performed the abdominal examination with the ultrasound machine. ANM C was engaged in supporting other ANMs (B and D). ANM C measured the blood pressure of client 1 and reported the value to ANM B. Then she measured the blood pressure of client 3 who was on the bench and recorded it in the woman’s handheld card. By the time she completed blood pressure measurement of client 3, client 2 sat on the bench, having finished her ultrasound. ANM C then measured the blood pressure of client 2. Then ANM C started asking client 2 if she has any problem and continued further examination and recordkeeping. [Field notes, Facility A, First visit]

In each of these facilities, ANMs exercised discretion in uniquely approaching ANC. ANC consultations were highly routinized, collective, and improvised, rather than structured as individualized, stepwise process as recommended by national guidelines [22]. The service provisions were different in terms of days or daily service provided in those selected facilities. For example, Facility C had designated ANC days where ANMs were under time pressure to examine all ANC clients in a single day. Facility A had more clients attending during a specific time (10 a.m. to 1 p.m.), which kept ANMs busy during this period. One of the reasons for having clients in this specified time was transportation problems in rural settings. Usually, ANMs spent more time (15–30 minutes) with women during their first visit to the facility, including performing the laboratory tests. For subsequent visits, if the women did not report any problems, ANMs spent only a few minutes (4–7 minutes) completing the examination, with slight variations between facilities.

*“It gets busy when all the clients come at once … Later, when the client flow is less, they have a lot of time, you might have also noticed they have enough time to chat … When clients come all at once, they are also in a hurry [and] won’t even wait … Sometimes, we don’t have any clients in ANC at all.”* (ANM E, Facility A, In-depth interview)

ANMs spoke of history taking and investing time in checkups as crucial components of ANC, although this was not practiced even when they were not under time constraints. However, there were a few instances (such as insufficient ANC staff or additional responsibility beyond ANC duties) where they were clearly overloaded.

“*While taking a family history, how is the family environment –supportive family or not? If there is time, we have to do the ANC checkup by utilizing a longer time and communicating, but it’s not like this. Here, you can see that one employee has a lot of work to do. It would be nice if we could at least give 10-15 minutes to examine at least one pregnant woman.”* (ANM R, Facility A, In-depth interview)

#### Contested scope of practice and rule-bending.

There was some contestation around the scope of practice of ANMs. This was notable in the extent of training and provision of ultrasound (USG). We observed debates over whether ANMs needed additional training to provide the initial ultrasound used primarily for calculating week of gestation. Doctors always carried out later abnormality scans that were often done at other facilities.

*“I have not received USG training. Even the doctor keeps telling me again and again not to do it because I am not fully capable. I have not received complete training, but I have been doing it. People come from far away to get free USG, and I feel pity for them, so I do it.”* (ANM B, Facility A, In-depth interview)

Maternal health services in government facilities are officially free of charge for service users in Nepal. Women are also given incentives to attend at least four ANC visits and to deliver in health facilities. No under-the-counter payments were observed; however, we observed that clients were charged for some aspects of care, including small or no charges (NPR 0–20) for registration (preparing ‘OPD’ card), NPR 350–675 for ANC blood test package (blood group, hemoglobin, random blood sugar, HIV, HBsAg, VDRL), and NPR 300 for an ultrasound scan when conducted by doctor. There also appeared to be some staff incentives to carrying out some procedures, such as: doctors for conducting USG, ANMs for dressing, conducting deliveries, post-natal home visits, and other staff (conducting lab test and x-ray) as clients paid for those services. Amounts were normally displayed in the facility, although they varied between facilities.

#### Interviews about and observations of EDSS use.

A clear disjunction existed between how ANMs spoke about the EDSS in interviews and how they used it in practice. Views on and use of the EDSS intervention differed between the formal interviews and observations, as summarized in Table 2. Generally, in interviews, ANMs expressed positive attitudes toward the EDSS, indicating that they understood what the EDSS was for, and they valued it. A staff nurse, who was rarely observed using the EDSS, explained how she valued its decision support prompts:

**Table 2:** ANMs’ response to EDSS with methodological differences.

Sub-themes	In-depth interviews	Observation + validation
Attitude towards EDSS	**•** All ANMs liked the idea of the EDSS in ANC **•** Almost all expressed the positive aspects of the application with very few technical problems	**•** Some untrained ANMs never showed interest to use in practice **•** Sense of irritation **•** Felt burden and pressurized to use
Perceived understanding and utilization of EDSS	**•** Explained the functions of pop-ups and their utilization in practice **•** Mentioned the importance of EDSS as decision support **•** Most of them verbalized EDSS helped them to provide all components step-by-step in ANC	**•** Utilized more as recordkeeping than decision support, ignored pop ups **•** No changes in following all components of ANC as recommended by EDSS
Valued the EDSS	**•** Verbalized the value or importance of EDSS	**•** They avoided using it (behavioral actions and excuses)
Time taken by EDSS	**•** Expressed the EDSS is time-consuming, required more time to use	**•** Sometimes spent 3–4 minutes, few times 15 + minutes
Continuity of EDSS implementation	**•** Almost all stated they would continue to use EDSS in future	**•** During the implementation phase, EDSS was not fully in practice

*“Many points are being missed when we do [ANC checkup] without using tablets. When we use the tablet, it reminds us, and if there is any problem, the tablet suggests advice. If the blood sugar is high, it suggests what to do further, and if there is a minor disorder like nausea, it suggests the statement about food [diet].”* (Staff nurse, Facility B, In-depth interview)

However, we observed very few times where ANMs used the EDSS for decision support; most used it as a recordkeeping application, if it was used at all. If the EDSS suggested that an ANM do something that was not what they usually did, they ignored it. So, for example, the EDSS would make prompts and recommendations for counselling or additional tests based on data entered, and ANMs were observed either ignoring EDSS prompts or entering that recommended counselling was done even when it was not performed. A range of explanations for nonuse or misuse of the EDSS were both observed and discussed. We observed ANMs not using the EDSS, with justifications like the client’s expected delivery date was close, the tablet was not available in the ANC room or not charged, or sometimes not using the EDSS when women visited the facility for not a full ANC visit (e.g., women attended either for conducting blood tests or having an ultrasound).

ANMs also commented that if they fully followed the instructions of the EDSS, which included all recommended aspects of ANC care, for example, counselling at every visit – it would make the ANC visits too long. ANMs explained that pregnant women were impatient to finish the visit and get home when transport was available. We observed some women walking for up to three hours from rural areas to the facilities, and we witnessed the problem of limited transportation or vehicles traveling on a specific schedule in the morning and evening. Women’s time constraints and transport limitations further reinforced pressure to shorten ANC consultations.

*“I have so much works left to do at home. The vehicle will be leaving soon [from the bus station]. Sister, please do my check-up quickly.”* (Pregnant woman, Facility A, Fieldnotes)

Some ANMs were frustrated by initial technical problems with the EDSS that resulted in the software being slow when the ANM wanted to enter information. Mostly, we observed the WHO EDSS tablets freezing when ANMs entered the value of test results, and this made them feel irritated about using the EDSS.

ANMs spoke about the increased recordkeeping workload imposed by the EDSS, which was implemented in this study alongside, instead of replacing, paper-based records. ANMs were observed having to enter, for example, vital signs or test results in the woman’s handheld record, the facility’s register and in the EDSS. The EDSS was not seen to help with recordkeeping, including for the computerized reporting of monthly services statistics to the district.

*“There is a thing that needs to be added in the app [EDSS]. What needs to be added is that we keep recording, but it would be good if monthly reports can be extracted from it. It would be better if everything done in the paper would be the same in this app and we don’t have to turn in papers and helps in online reporting.”* (ANM B, Facility A, In-depth interview)

During our first visits, when EDSS had not yet been implemented in these facilities, four ANC protocol visits were performed. However, by the third visits, during the implementation of EDSS, these facilities started following the WHO recommendation of eight protocol visits, endorsed by the Nepal government countrywide. All ANMs tried to comply with the government’s protocol, in contrast with EDSS use. Generally, they viewed EDSS implementation as a temporary change and more of a research project. Although there were a few times where clients had difficulty complying with the eight ANC visits due to the rainy season and transportation issues, ANMs were flexible in calling clients for follow-up visits, considering their difficulties.

*“This app is separate; now it’s not in the government protocol, and we are operating it from a non-governmental organization. We must send a report to the municipality. We are afraid that the details might get missed in the register, so we have to maintain our register first.”* (ANM J, Facility C, In-depth interview)

In informal conversations, and in interviews, ANMs spoke about whose responsibility it was to use the EDSS. The mIRA project model was that one person from each facility was externally trained to use the EDSS and given the tablet for the facility. Onsite support by fieldworkers in the first month of implementation was intended to support both the trained ANM and untrained ANMs in using the tablets and software. The interviews revealed that some untrained ANMs felt that it was the responsibility of the person who had been trained to use the EDSS, but it was not their responsibility, or they had not had sufficient training. We observed similar tensions around other trainings that took place in the facility: the person who was trained was expected to implement the practice and this was not necessarily seen as the responsibility of all ANMs.

*“We have a mindset. Even I follow this mindset that, who went for training should be responsible and should work on it.”* (ANM G, Facility B, Informal conversation)

*“Tablet is being used [by] who have received the training, it is the focus for her [EDSS trained ANM].”* (ANM I, Facility B, Informal conversation)

### Part 2: Street-level bureaucracy theory and strategies in ANC delivery

#### Belief that they were providing good quality of care.

The service delivery practices were underpinned by ANMs’ beliefs about what constituted “good quality” ANC, which we analyzed using street-level bureaucracy theory. In both formal interviews and informal conversations, ANMs consistently perceived the ANC they provided as good quality, often equating quality with service availability such as USG and laboratory test, rather than complete clinical assessment or counselling as recommended by guidelines. ANMs also explained that they were seeing relatively few women and those that they saw were generally in normal condition. If there was any sign of any issues, then the women were immediately referred either to a doctor working in the facility or the nearest hospital.

When we interviewed ANMs about their perceptions of the quality of ANC, they emphasized the importance of maintaining women’s privacy. However, during our observations at Facility A, we observed that up to four women were examined simultaneously in the same room, including one seeking a termination of pregnancy. Although ANMs defined quality in terms of privacy and respectful care, observations revealed a gap between these ideals and actual practice.

*“We should clear their confusion regarding the quality of services being provided or not. Another thing is, we should maintain their confidentiality. Some of the women might be in fear due to family pressure. We should create an environment for them to share their problems without any fear. I think this is also quality service.”* (ANM B, Facility A, In-depth interview)

Most women seemed satisfied with the ANC services provided when we enquired. However, we observed that ANMs often missed components of ANC, such as complete history taking, head-to-toe and abdominal examinations, proper counselling and basic tests that should be performed during relevant visits (such as urine test, hemoglobin test, blood sugar test, etc.).

“*It is much better now. Previously ANM were old and they didn’t used to communicate properly and didn’t provide any advice, they just used to provide medicines but now these ANM are different. They* *provide us information about what I asked, provide appropriate advice and listen to us*.” (Pregnant woman, Facility A, informal conversation)

#### ANM behaviors to move towards and away from ANC clients.

Our observations revealed that ANMs exhibited behaviors that diverged from ANC guidelines, including those recommended by the mIRA project EDSS intervention or from the government’s new eight ANC visits policy. Using street-level bureaucracy theory, we explore how ANMs adapted the intervention or policy to suit ANC clients (moving towards) but also to suit themselves and their values and context (moving away) and rationalized these differences (Table 3). ANMs rationalized routine ANC practice, categorized or prioritized the clients, or distanced themselves from care provision. For instance, ANMs in these facilities generally prepared the woman’s handheld ANC card after three months of pregnancy. Prior to the end of the first trimester, ANMs used OPD cards to record information. ANMs reasoned that some pregnancies were not wanted and would be aborted, so the woman’s handheld card was not needed. However, in one instance, an ANM wanted to skip preparing the woman’s handheld card even after three months and was observed attempting to convince the client to visit a nearby health post, which was closer to the woman’s home, though the woman wanted to seek ANC in that ANM’s facility (Table 3, example 5). In some cases, ANMs considered the financial implications for women from poor economic backgrounds when performing investigations, if the women appeared normal (Table 3, example 2). Moving away behaviors reflected other strategies such as over-referring minor abnormalities to doctors or higher centers (Table 3, example 7) or skipping routine checks like blood pressure measurement (Table 3, example 8). These practices show how ANMs rationalized their actions to suit their own context. The ultimate implications were that they compromised on providing good care, tried to short-cut routine practice, and did not provide equal care to every pregnant woman.

**Table 3:** ANMs behavior: Moving towards and away from ANC clients.

Antenatal service provision	Examples of behavior	SLB strategies	Adaptive process	Implications on care provided
**Moving towards ANC clients**	1. The senior doctor started to charge for USG when performed by him. Because of this system, ANMs tried not to send women to pay for the ultrasound if found normal	Rule bending	Untrained ANMs learned to perform USG from the USG-trained ANM and provide the services Change back the rule by the doctor The doctor helped ANMs in performing USG when there was difficulty	Financial benefits to the clients Some efforts were made to provide care that is beneficial to the clients.
	2. Some ANMs requested women to have ultrasound scan from the doctor if they were unable to go to the higher center for anomaly scan	Rule bending	ANM attempted to ensure that at least some abnormalities could be identified if examined by a doctor present in the facility, with a minimal charge	
	3. Some ANMs provided flexibility to the recommended follow-up dates when it was challenging for rural women to visit the facility 4. One ANM found her relative’s blood pressure was not measured by another ANM. She brought the woman in the ANC room and measured her blood pressure	Rule bending		
**Moving away from ANC clients**	5. One ANM tried to skip preparing the woman’s handheld card from the facility, instead requesting the woman visit the health facilities nearby the client’s home 6. Some ANMs waited for women to be at least 12 weeks pregnant before preparing the woman’s handheld card	Rationing practice Distancing Routinizing practice	Gaming the system	ANMs examined the clients quickly and compromised in providing good care
	7. ANMs referred to the doctor or higher center in case of minor abnormalities	Invoking different policy understanding	Risk-averse	ANMs did not provide equal care to all clients
	8. One ANMs frequently missed measuring the blood pressure of ANC clients	Rationing practice		ANMs referred ANC clients than clinically required
	9. Some ANMs took shortcuts while providing antenatal care services and were involved in other services	Client categorization Distancing		
	10. ANMs made judgments about when or whether to provide information or counselling to the women	Client categorization Prioritizing		

Overall, these practices demonstrate how ANMs exercised discretion in balancing formal guidelines, EDSS recommendations, and perceived client constraints. While such adaptations were often justified as protecting women from financial burden or unnecessary procedures, they also resulted in selective implementation of ANC components and inconsistent adherence to standardized care pathways. These discretionary practices reflect classic street-level bureaucratic coping strategies, with implications for equity and quality of ANC delivery.

The content and extent of counselling in ANC visits were adapted based on perceived need. This further illustrates how discretionary judgment, rather than algorithmic guidance in the EDSS, shaped ANC service delivery. Generally, ANMs, instead of giving the same information to all women, made judgements about what and when to give information to ANC clients. ANMs explained that in early ANC visits, when preparing the woman’s handheld card, they interacted with women more because they had to ask more questions. In later visits, ANMs focused on calculating gestational age and checking weight, blood pressure and fetal heartbeat, and only offered counselling if women complained of any problems.

“*If women do not have any problems, then what should we explain to them? They are all okay. In the initial visit, we asked about having headaches and blurred vision and then counseled them to visit facilities as soon as possible; in the other visits, if they did not complain about any problem, we felt that counselling was not required*.” (Validation meeting, Facility C)

Care provision was also shaped by ANMs’ perceptions of risk and liability. ANMs often referred women when ANMs identified minor complaints such as nausea, diarrhea, or back pain that could be manageable at their level. ANMs did not want to take a risk if a doctor was present in the facility, or they tended to refer pregnant women to higher-level facilities. When discussing caring for complications, ANMs stated that these risks depended on circumstances. Some ANMs did not want to take the risk if higher-level facilities were nearby, but in more remote areas, if doctors or a higher-level facility was not accessible, ANMs would have to take the risk and manage these more minor complications. Also, in some scenarios, pregnant women preferred to be referred to higher-level facilities for minor complications.

“*Sometimes clients do not want to be referred; sometimes they choose to be referred; it is their right to choose; sometimes clients are so complaining that we are threatened, if something happens, then they will create a hassle in the facility and damage things*.” (Validation meeting, Facility D)

The ethnographic work revealed how social structures and entrenched practices shaped provision of ANC and how little the EDSS intervention did to alter these systems. The research participants recognized that the system that healthcare providers were used to was shaping their behavior and resulted in the lack of effect of the EDSS on changing practice. One facility-in-charge explained:

“*The healthcare providers are operating with a 19th-century mindset in a 21st-century system. They continue to practice as they always have and do not attempt to change because they believe this is simply how things are done. Changing both the prevailing mentality and the system is difficult*.” (Validation meeting, Facility D)

## Discussion

This study examined how ethnographic facility case studies, conducted as part of a process evaluation [19], revealed critical disconnects between desired practices and on the ground realities, demonstrating the value of ethnographic observations for evaluating EDSS implementation in ANC. Our study highlighted how ANMs responded to the EDSS intervention, finding similarities with how SLB theory characterizes the gaps in policy implementation by frontline staff and drivers of those behaviors, and how it ultimately affected the quality of care [37,38]. The theory of street level bureaucrats has been widely used in health system literature to explain and theorize how frontline health workers implement policy in ways that differ from what policy implementers propose. The ways health workers provided care were based on their own logic, circumstances and values, rather than acting as ‘angels or robots’ who blindly follow orders [39]. Some of the divergence was beneficial to ANC clients, but some was less beneficial and negatively impacted on the quality of ANC.

### Discrepancies between rhetoric and practice

In this study we illustrated the differences between what was said and what was done (sometimes characterized as the ‘know-do gap’). The different data collection approaches used in this study explored how the EDSS was implemented in reality. A multi-method ethnographic approach, with multiple modes of data collection over time, revealed discrepancies between the rhetoric and practice of how ANMs discussed and enacted their practices, particularly in response to formal guidelines for providing antenatal services in primary health facilities. For example, ANMs conveyed knowledge of good ANC practices in formal interviews, which conflicted with our observations of their actual practices. During informal conversations throughout our observations, ANMs revealed their challenges with the EDSS, contrasting with their positive views about EDSS expressed during formal interviews. Generally, ANMs reported a consistently positive attitude towards the EDSS and its use, despite experiencing technical frustrations and time constraints, though this did not translate into use [26]. We often observed ANMs having more time to dedicate to providing ANC, except in specific situations of higher client load, but that their practices did not change.

We found that relying on only one method could lead to conflicting conclusions about the response to and impacts of EDSS intervention. The literature also suggests that longitudinal qualitative approaches embedded with case studies and ethnographies unpack the complexities of healthcare interventions and illuminate real changes in outcomes over time [29,40–42]. Most evaluations acknowledge only simplistic differences between contexts where interventions are implemented; however, Hawe and colleagues have argued for exploring ‘more naturalistically’ how interventions might vary across different community contexts [43]. Exploring and understanding the social and organizational settings of interventions is a valuable role of ethnography in evaluation.

### Challenges in changing systems

We approached the work without assuming that health workers are ‘angels’ motivated by nothing but altruism to serve the patients, or ‘robots’ who do what they are blindly instructed to do by above [39]. Instead, they are individuals with multiple identities, working in large bureaucratic and hierarchical health systems, who have experience with many research projects and interventions that have come and gone, and they have responded to interventions in a context where many factors are constantly changing. We used SLB theory to uncover the realities of ANMs’ behaviors and actions in providing antenatal services [37]. Our study provided insights into ANC provision and how ANMs responded to the implementation of ANC guidelines, including those recommended by the EDSS. The EDSS was implemented by ANMs, but not in the way the evaluation intended or hoped would result in meaningful changes in the quality of ANC [23,26]. The EDSS recommendation to improve the quality of ANC did not fit well into ANMs’ practice due to the reality of ANC structures in the setting. Contextual factors, such as the way ANC was organized with ANMs mostly working together, rationalizing or rationing care for individual women, or varying time constraints in ANC workload influenced ANM’s ANC provision and EDSS utilization as intended [26]. Moreover, we believe that technical issues related to the version changes of the EDSS, along with freezing issues, also affected its optimal utilization [24].

As highlighted by SLB theory, the frontlines do not automatically comply with all imposed policy mandates, rather, they practiced a version of the mandate that fit with their context and day-to-day realities [34,44]. The changes in health facilities over time, including availability of services, staff cuts, workload, and political shifts, may also have impacted ANMs’ use of the EDSS in these facilities. Moreover, the way ANC is provided in these settings has been established over a long period and has been adapted into their system; as a result, integrating EDSS into their workflow was a challenge for them. Therefore, integrating the EDSS into the existing government reporting system and adopting task-sharing approaches were emphasized by ANMs to reduce the recordkeeping burden and support the use of EDSS in daily practice [23,24]. Adaptive health system conditions such as organizational capacity, resource availability, policy implementation, technological experience, and network connectivity shape the use of digital health interventions, like the EDSS in our study [45,46].

Challenges arise when implementing new programs or standards without considering current workflows, practices, and factors that hinder the delivery of quality care [47]. We observed ANMs adopting the WHO’s eight protocol visits, endorsed by the Nepal government, during the implementation of EDSS, although there were a few instances for non-compliance. They made efforts to implement this change from a legitimate source. ANMs perceived it as permanent change, contrasting with our temporary EDSS research project, which was implemented for around 6 months. However, our findings also revealed gaps in fully implementing the WHO eight protocol visits by ANMs due to factors such as the unavailability of physical resources, geographical and transportation constraints, and personal and financial obstacles faced by women to attend the recommended visits.

Through this study, we found that ANMs perceived themselves as examining healthy women, as they encountered very few complicated cases. Further, when ANMs did face any complications, even if minor, most of them were risk-averse and reluctant to provide care outside of routine cases. The fact that ANMs did not try to take risk was due to the presence of medical officers or doctors in these facilities and access to higher centers and client’s preference for referrals. The EDSS was meant to guide ANMs’ decisions for the problems they encountered at their level as it followed Nepal’s reproductive health protocol and WHO guidelines, in the mIRA and WHO EDSS, respectively [48]. Rather than following guideline-informed EDSS recommendations, ANMs preferred referring clients. This is unlike other settings where a study conducted among primary care doctors in India identified them as being risk-averse due to unavailability of staff, resources and facilities to deal with emergencies, as well as trust issues of patient [36]. In our study, ANMs thought that they were already providing good care based on their level, and quality was not a problem. This shaped their behavior in ANC and in utilizing EDSS care recommendations and indicates a potential gap between providers’ perceptions and the actual quality of care delivered.

### Strengths and limitations

Both primary researchers (SK and SD) for this study were born and raised in Nepal. Through frequent visits and informal interactions with ANMs and facility staff, as well as sharing a similar cultural background and language, the researchers developed rapport with participants which enhanced the data collection and validation process. We developed good, trusting relationships with them and eventually, ANMs felt at ease to share information and their on-the-ground realities related to ANC within the facilities. Additionally, the researchers for this study were not involved in the collection, or aware of results from, data from other studies in the evaluation, such as pre-post implementation and monitoring data.

However, the study was not without limitations. We were limited by time and resource constraints for how long we were able to conduct data collection, within each visit and overall, for each facility. Additionally, our (researchers) movement between different facilities may have caused us to miss emerging behaviors or events that occurred in our absence, potentially limiting our comprehensive understanding of ongoing dynamics. We tried to address gaps in our knowledge through the validation meetings, where preliminary findings were discussed and cross-checked with both facility staff and fellow researchers. We explored, to a limited extent, the perspectives of clients receiving ANC services before and after the implementation of EDSS. Future studies could incorporate more in-depth exploration of clients’ perspectives to better understand their experiences and perceptions of care provided through EDSS. Finally, this study was conducted among four government facilities – two PHCCs and two HPs – with one of each type implementing the mIRA EDSS and the other implementing the WHO EDSS to ensure representation across facility types. We selected better-functioning facilities with higher ANC volumes because low-functioning or low-volume sites were likely to have limited use of the EDSS, which would have reduced our ability to capture meaningful insights into real-world implementation and user engagement. Low-functioning or low-volume sites may face further implementation challenges that our study was not designed to or able to uncover. The findings might not be fully generalizable or reflective of the experiences of other facilities in the mIRA project evaluation, or of other primary health facilities in Nepal. Future research could include facilities with varying performance levels and service volumes, including peripheral and lower-resourced sites, to capture a wider range of implementation experiences.

## Conclusion

This study provides both methodological insights and empirical evidence on the implementation of EDSS for ANC in Nepal. Our findings reveal that the use of the EDSS was shaped more by the everyday realities of ANMs. As street-level bureaucrats, ANMs exercised substantial discretion in how they delivered ANC and engaged with EDSS, routinely adapting, bypassing, repurposing its recommendations to align with existing workflows, time constraints, perceived risk, and client expectations. Some adaptations aimed to reduce burden on clients, while others resulted in incomplete or inconsistent adherence to guidelines. The EDSS was largely used as recordkeeping tool rather than for decision support, reflecting a lack of integration with the health information system, parallel paper-based reporting requirements, technical challenges and the perception of the EDSS as a temporary intervention, which further reduced its legitimacy and uptake.

These findings suggest that EDSS alone are unlikely to improve ANC quality unless they are designed and implemented in ways that explicitly account for the realities of primary healthcare settings. EDSS intervention designers should consider how prompts may conflict with existing practices and beliefs and anticipate how users are likely to repurpose recommendations. This may involve an iterative balance between guideline-derived algorithms and acknowledgement of the drivers of common practices when tailoring prompts for behavior change. Ethnographic approaches can support in-depth contextual understanding to facilitate these adaptations. EDSS must be embedded within health system structures, aligned with existing workflows, and designed to acknowledge providers’ discretionary power and coping strategies. Implementation strategies should also consider staffing patterns, resource availability, ongoing technical support, client load, and geographical challenges that influence ANC delivery. Providing regular refresher training that address ANMs’ confidence, perceptions of clinical risk, and risk-averse behavior is critical to fostering behavior change and encouraging adherence to guidelines.

This study contributes to a detailed evaluation of implementation realities, emphasizing the value of an ethnographic, multiple methods approach to unpack contextual factors and reveal gaps between rhetoric and practice. By triangulating methods, it provides a nuanced view of ANC provision and the challenges encountered in efforts to improve the quality of care.

## Supporting information

S1 Text Description of EDSS intervention and workflow. (DOCX)

S2 Text Observation and interview guide. (PDF)

S3 Text SRQR checklist. (DOCX)
