# Supplementary material for: The implementation realities of a digital antenatal care improvement intervention: Insights from ethnographic work in primary health facilities in Nepal
Source: PLOS Digit Health. 2026 Apr 6;5(4):e0001340. doi: 10.1371/journal.pdig.0001340 (PMC13052878; doi:10.1371/journal.pdig.0001340)
Supplement: S1 Text — (DOCX) [file pdig.0001340.s001.docx]

**Description of Electronic Decision Support System (EDSS) Intervention and Workflow**

**Training workshops**
After consent process in all primary healthcare facilities, the intervention was initiated through two three-day, in-person training workshops (one for the mIRA EDSS and one for the WHO EDSS) held at Dhulikhel Hospital. One auxiliary nurse midwife (ANM), nominated by the local municipality, attended from each participating facility. The training workshops covered the aims of the mIRA project, use of tablets and EDSS software, and hands-on data entry practice with support from project staff. Trained ANMs were expected to cascade the training to other ANMs at their facilities. Training also included use and interpretation of oral glucose tolerance test kits, which were distributed to all facilities as they were not routinely available and were recommended by the mIRA EDSS.

**On-site support**

Upon training completion, each facility was provided with a tablet installed with allocated EDSS and glucometers and test strips to facilitate performance of oral glucose tolerance tests. A project fieldworker provided on-site support at each facility for a 3–4-week lead-in period to assist both trained and untrained ANMs in using the EDSS during ANC consultations. In facilities with low patient volume, ANMs practiced using dummy data or information from previous ANC visits.

**Hardware and software**
Each facility received a Samsung Galaxy Tab A 8.0 tablet preloaded with the allocated EDSS software. Tablets were supplied with SIM cards for mobile internet access, although connectivity was variable in some areas. Devices were password protected, with restricted access to backend databases. The WHO and mIRA EDSS had similar functionality but differed in user interface and dashboard design: the WHO EDSS used a more colorful interface, while the mIRA EDSS mirrored the layout of Nepal’s paper-based ANC card. The flowchart of mIRA EDSS is presented in Figure 1 and WHO EDSS in Figure 2.

Both EDSS prompt sets and data entry forms were aligned with Nepal’s antenatal care guidelines—the Reproductive Health Clinical Protocol for Staff Nurses and ANMs—as well as the 2016 WHO guidance on routine antenatal care. However, the software differed in emphasis, particularly in relation to pregnancy complications. The WHO EDSS primarily focused on screening and referral, whereas the mIRA EDSS offered tailored diagnostic and treatment prompts for gestational diabetes, hypertensive disorders of pregnancy, and anaemia. Prompts related to routine components of antenatal care were largely similar across the two EDSS. For instance, both systems included a ‘counselling’ icon on each dashboard. Selecting this option opened a data entry form listing counselling topics such as diet, danger signs, institutional delivery, and breastfeeding, with content aligned to Nepal’s antenatal care guidelines. As ANMs clicked on each counselling topic, they were prompted to provide the corresponding information to the woman.

After the supported lead-in period, additional technical support was provided through separate WHO EDSS and mIRA EDSS Viber groups that included ANMs from participating facilities. Two major technical issues arose during implementation. In WHO EDSS facilities, limited tablet memory caused freezing and slow loading; higher-capacity memory cards were therefore provided mid-implementation. In mIRA EDSS facilities, a software bug prevented ANC visit entries from syncing to the cloud server, leaving data stored only on the tablets. This issue was resolved, and data synced part-way through implementation, while few facilities continued to experience persistent syncing problems.

**mIRA EDSS Workflow and Dashboard**


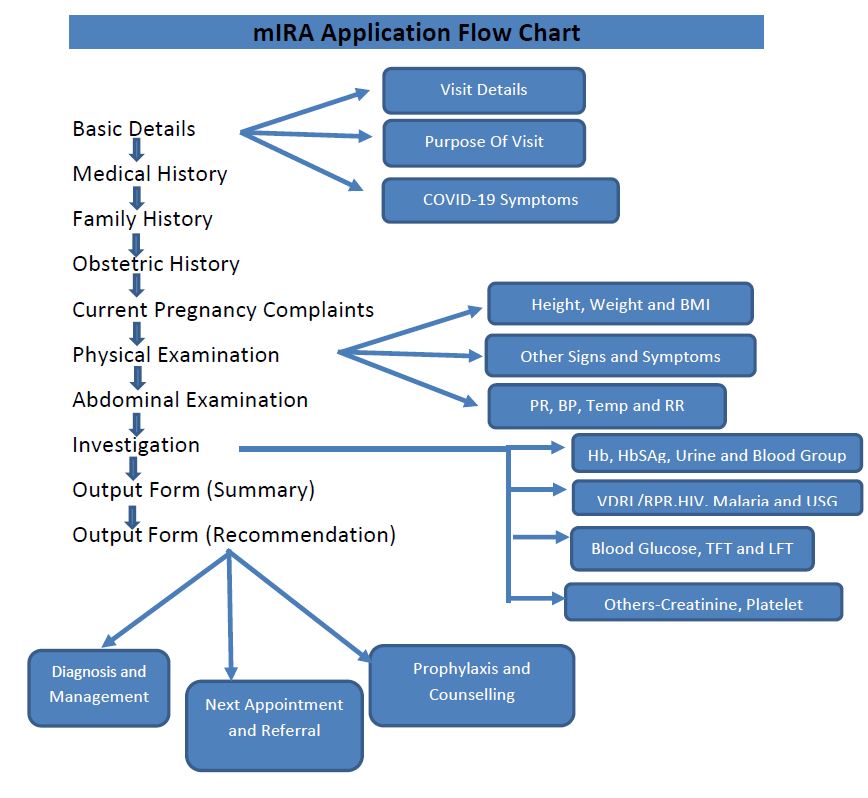

*Figure 1: Screenshots of the mIRA EDSS with the (left) screen showing the dashboard menu and the (right) screen showing an example data entry form*

**WHO EDSS Workflow and Dashboard**


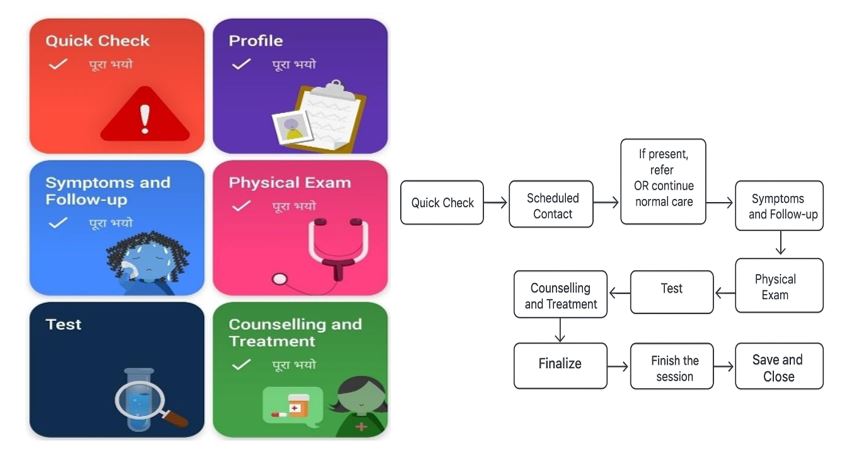

***Figure 2:*** *Screenshots of the WHO EDSS with the (left) screen showing the dashboard menu and the (right) screen showing an example data entry form*

**Reference**

1. Supplement to: Karmacharya BM, Das S, Shrestha A, et al. A novel approach to assessing the potential of electronic decision support systems to improve the quality of antenatal care in Nepal. *Glob Health Sci Pract*. 2025;13(1):e2300370. <https://doi.org/10.9745/GHSP-D-23-00370>
