# Supplementary material for: The implementation realities of a digital antenatal care improvement intervention: Insights from ethnographic work in primary health facilities in Nepal
Source: PLOS Digit Health. 2026 Apr 6;5(4):e0001340. doi: 10.1371/journal.pdig.0001340 (PMC13052878; doi:10.1371/journal.pdig.0001340)
Supplement: S2 Text — (PDF) [file pdig.0001340.s002.pdf]

Work Guide for the Longitudinal Case Studies - Nepal

Visit One – Before The Start Of The Intervention

Facility as a whole:

1. Map the facility – i.e., the physical layout of the clinic - and what is done where
2. List the staff in the facility, and their jobs/qualifications, and their responsibilities, and how long they have worked at the facility
3. Document the schedule of the facility. I.e., how is care organised. What services are provided on what days. I.e., ANC days, immunisation, well baby, NCD care etc Routine outreach work, visits from other health personnel etc.
4. Explore with staff what they feel the challenges that they are facing as a clinic. What they are doing well. What they would like to do better. What is frustrating ? What would they like to do differently that they can't.
5. Explore with the clinic in charge what are the various programmes and projects that the clinic is currently involved in. What are their reporting requirements? What happens with these reports they send? Do they get feedback? Is there any regular supervision of the clinic? If so what does it entail. What aspects of care do they think that their supervisor cares about?
6. Draw a diagram of the clinic and its referral networks and linkages. I.e., do they supervise any facilities? Do they do any regular outreach to facilities. What about community health workers. Where do they refer to if there are issues? Who do they phone if there is an issue. Who do they work with at the municipality.

ANC services particularly:

7. Map how women move through the facility to receive an ANC visit. And what is done where by who. I.e., where does she wait, where is her blood pressure taken and by who, where are the blood tests done and by who? Where is the physical exam done and by who. Time how long ANC visits take.
8. Document how much the providers involved in the woman's care interact with the women, and the kind of areas they interact on, and the quality of that interaction.
9. Note down what is recorded where and when for ANC visits. When the various records are filled in by who.
10. List the order in which elements of ANC care are provided.
11. Discuss with staff why things are organised the way they are. Is this similar to how they were trained? Have they worked in other facilities where it was done differently. What are the considerations that took into account when organising the care. I.e., not wasting staff time, not wasting women's time, what is convenient? What can be done by more junior staff? Which staff are most busy?
12. Explore with staff their perception of the quality of ANC care. What they think goes well. What are the challenges they face.
13. Explore/describe how they work with the referral facility in ANC care .

14. Explore with staff if they feel that there are some aspects of ANC care they would like to know more about, that they feel should be improved, that are missing in the current practice.
15. Explore with staff what they feel are the issues that that women face using ANC? What they think women appreciate? What women complain about? Why they come or don't come for ANC services.
16. **During this visit the time motion study will take place. Please see separate tool.**

Visit Two – relatively soon after the EDSS starts being used at the facility

The main focus of this visit will be to understand how they are adapting to using the EDSS.

1. Who went for training. What did they think of the training? What did they like most about the training? What did they learn? When they were having training could they see how the EDSS would help. What problems did they worry about.
2. How has the onsite support worked? Has it been useful? What were the kind of problems that staff were experiencing. Was it getting easier to use the EDSS. Were there anticipated problems that had arisen. Were there elements in the EDSS that helped them provided better care? Was anything missing from the EDSS. How do people talk about the EDSS
3. Using the work that you did in the first visit mapping where ANC care was provided, and by who, and the order in which ANC care was provided – explore if this has changed when staff use the EDSS. Map / note down any differences. Discuss this with staff.
4. Observe and document if you think how providers interact with patients has changed when staff are using the EDSS. Do you think it increases or decreases the interaction with women? If you see any differences discuss this with staff.
5. Observe if women say anything / ask about the EDSS or if they are told about it.
6. Observe/explore/describe if it changes the way that health workers interact with women. If you see any differences – discuss this with staff when women have left.
7. Observe/describe if you see any differences with the record keeping / reporting/ supervision that was documented in the first visit.
8. Describe if there have been any changes to the overall running of the clinic documented in the first visit.
9. Document if there are other programmes or changes in the clinic that have happened independent of the EDSS.

Visit Three – Towards the End of the Trial

1. Explore/discuss what is happening with the EDSS use in the clinic. Is it still being used in the way that was anticipated.
2. Discuss ow has EDSS been integrated into care?

3. Explore /discuss / compare what you have documented about the organisation of ANC care in the previous two visits – and whether things have resorted to how they were before, whether they are still as they were in the second visit, or whether more changes have been made.
4. Discuss with staff how they think that provision of ANC care might have changed due to the EDSS.
5. Observe if the changes in how health workers interact with women has changed since you last visit.
6. Observe/document if there have been changes in reporting, supervision on other elements of care since your last visit.
7. Discuss/document what are the overall issues facing the clinic at that time independent of the EDSS.
8. **During this visit the time motion study will be repeated. See related Time-Motion Checklist.**
9. **During this visit – formal IDIs will take place with ANMs working in the facility. See related Interview Guide.**

Visit Four

1. Feedback to staff your preliminary findings on the implementation of the EDSS. Discuss with staff whether they feel that it is an accurate understanding of what happened. Ask them if there is anything that they feel that the research team missed, miss understood, or got wrong – as well as what they agree with.
2. Work with the staff to develop their feedback to the research team on the EDSS and its implementation. Whether they would recommend roll out. What worked and what didn't.
3. Work with the staff to develop a presentation/document on any other issues that they feel they would like to raise with the municipality/researchers /Dulikhel etc.

Telephone Interviews – between facility visits

1. How have things been going in the facility in the last two weeks?
2. What have been the issues/tasks you have been dealing with/focusing on at the facility in the last two weeks?
3. Has anything happened related to ANC care in the last two weeks?
4. How have things been going with using the EDSS in the last two weeks?

Final engagement with facility

Bring together in charge or other staff from all three sites where the longitudinal study took place to discuss similarities and differences in the experience and their feedback on implementation of the EDSS. Prepare a joint presentation to the research staff/policy makers/health officials.

Interview Guide for Auxiliary Nurse Midwives (ANMs)

Name of Facility: ____

Name of Participant: ____

Before the start of the interview, please seek written informed consent for participating in the interview and for audio recording of the interview. Kindly reiterate that participation is purely voluntary and the information that they provide will be kept confidential with access to only the study investigators. Please also inform that their names will not be revealed while reporting the results. Please let them know that the interview may last for 30 minutes. Please ask the interviewee if they have any queries and clarify them before proceeding with the interview.

Before starting the audio recording, state "Audio recording started".

ENSURE THE INTERVIEWEE HAS SIGNED THE CONSENT FORM BEFORE COMMENCING THE INTERVIEW

Please note that this a working guide to help develop and pursue questioning in the interview and only represents the main themes to be discussed with the participants.

Introduction

Could you start by telling me a little bit about yourself and how long have you been working at this facility?

Could you tell me about your responsibilities at this facility?

What experience do you have with using tablets or mobile devices?

> **Prompt**: Do you have a personal mobile that you use to access the internet (eg a smart phone)?

Workflow in ANC

Can you tell me about how ANC is provided at this facility? Talk me through a typical day of providing ANC?

> **Prompt**: What do you do? What do your colleagues do?
>
> **Prompt**: What do you do at the time that the patient is with you – and what do you do afterwards?

When a patient comes into the facility for ANC care, what information needs to be recorded and where? Probe for all places (ANC register, individual patient records, etc).

> **Prompt**: If you need to know something from a previous visit of the woman, how do you find out? Where do you look?

Impact of mIRA/WHO EDSS

Can you tell me about your experience of using tablets for ANC in this facility? Are you still using it?

Prompt: What do/did you like about it? What don't/didn't you like?

How do you use the tablets for ANC? How does it fit into your workflow:

Prompt: For example, do you fill in the tablet while you are examining the pregnant woman? Or do you use it after the ANC visit is over?

Do you think the tablet has changed ANC is given at this facility?

Prompt: For example, have you changed the order in which you do certain tasks?

Prompt: Do you share or split tasks with any of your colleagues?

How has the tablet changed how much time you spend performing your ANC duties?

Prompt: For example, do you think you spend more or less time checking pregnant women during their visit? What about time for maintaining records?

Conclusion

Is there anything else that you would like to comment on that I haven't already asked you about?

Thank the interviewee for participation. At the end of interview state "Audio recording ended".
